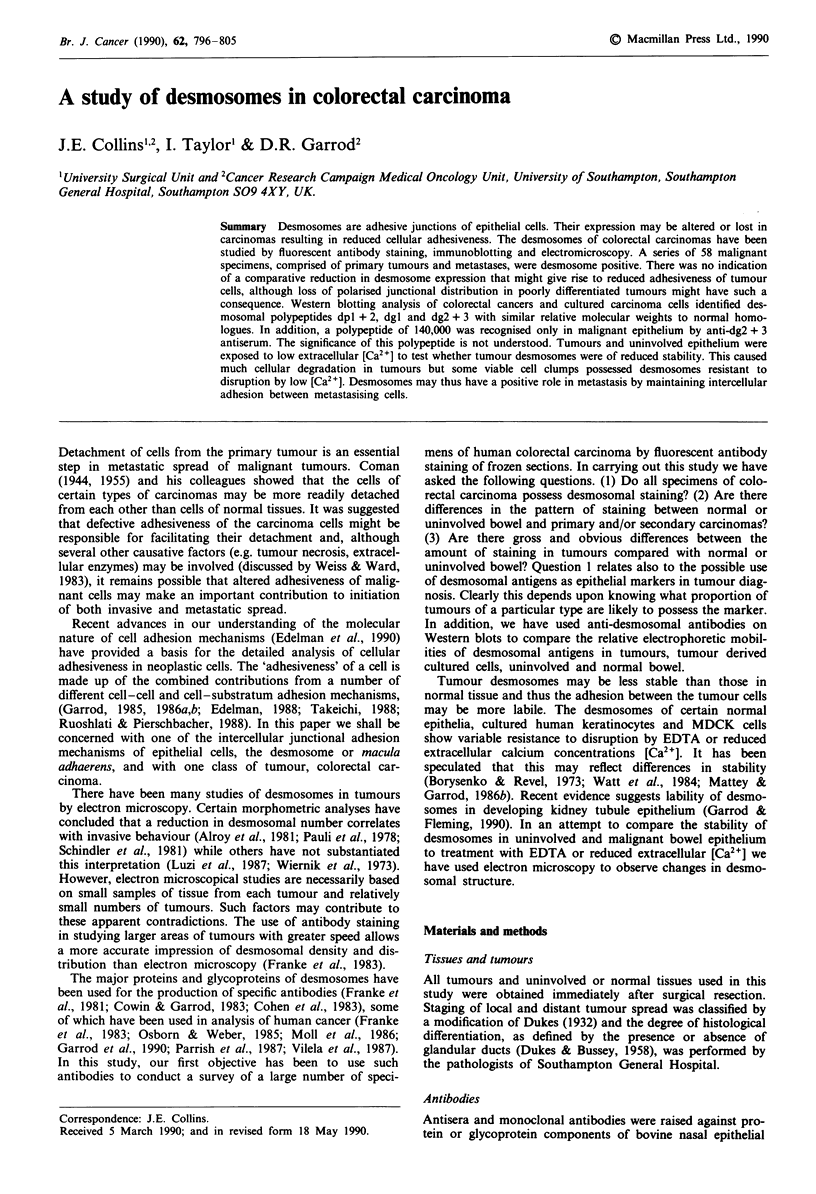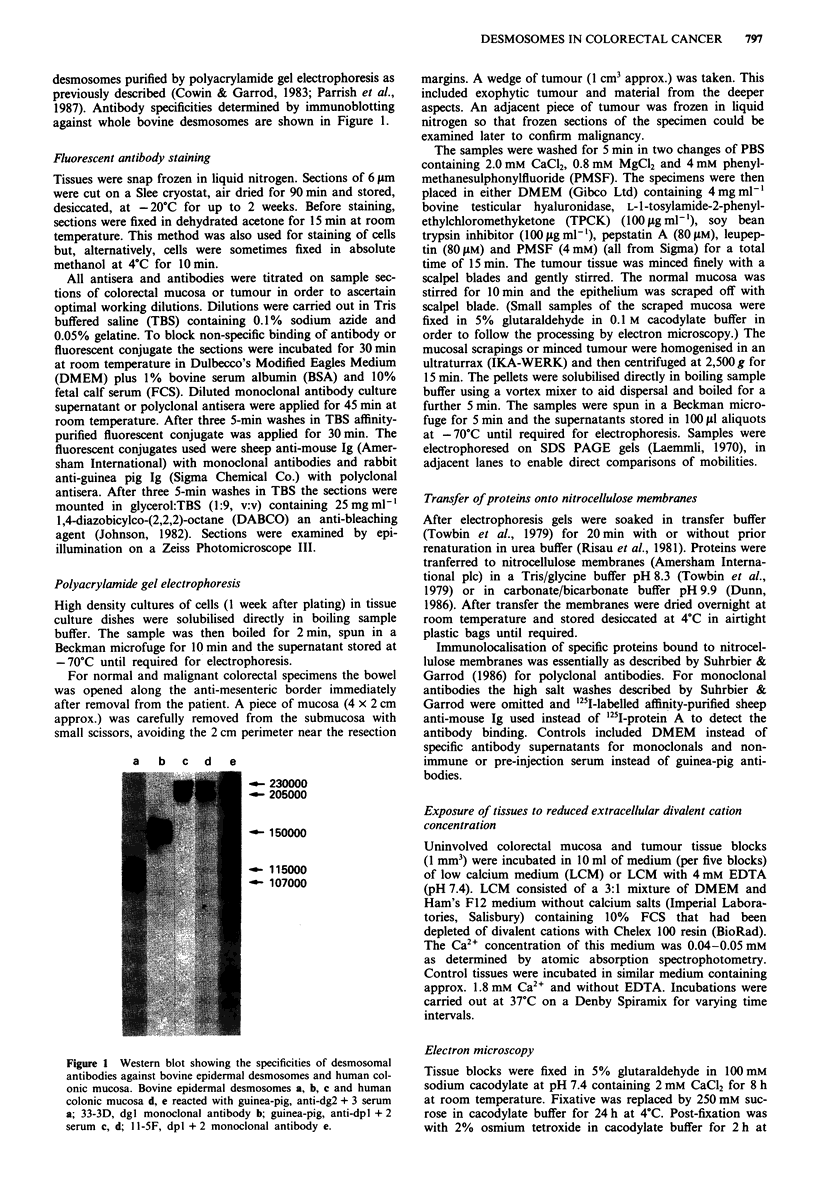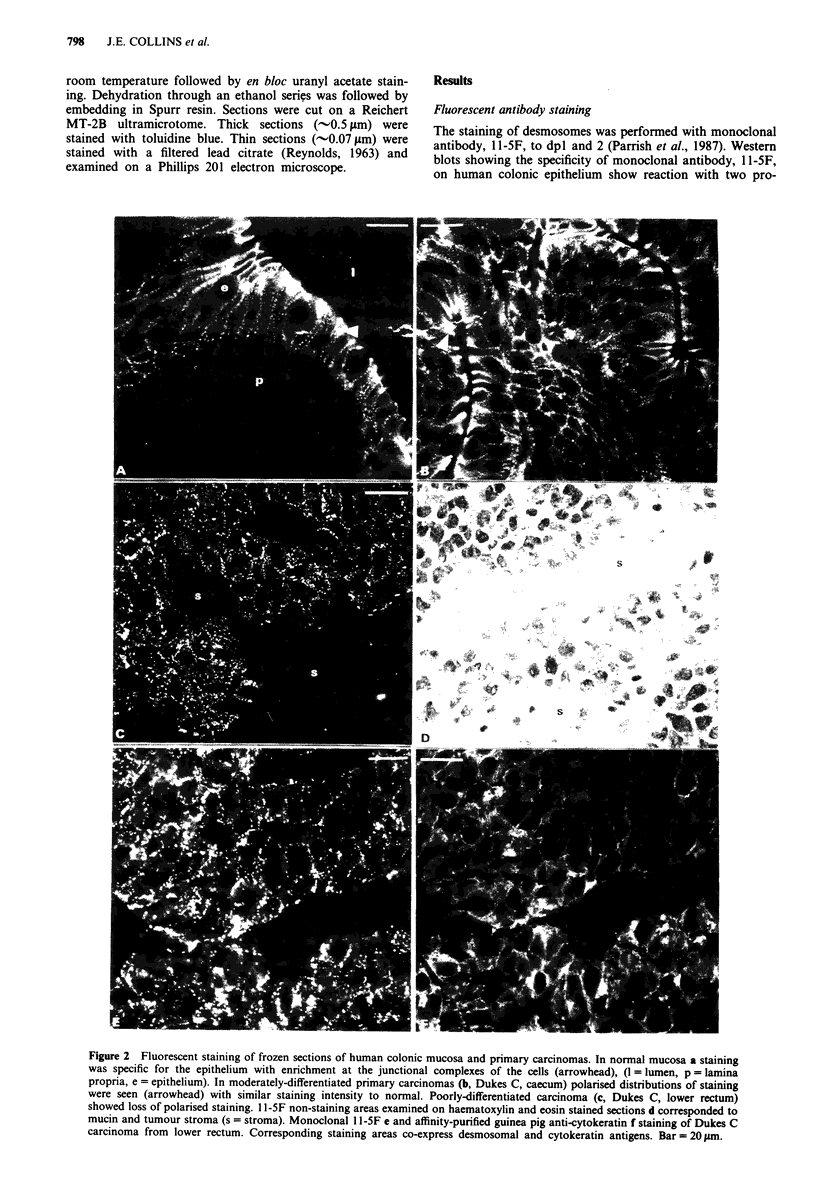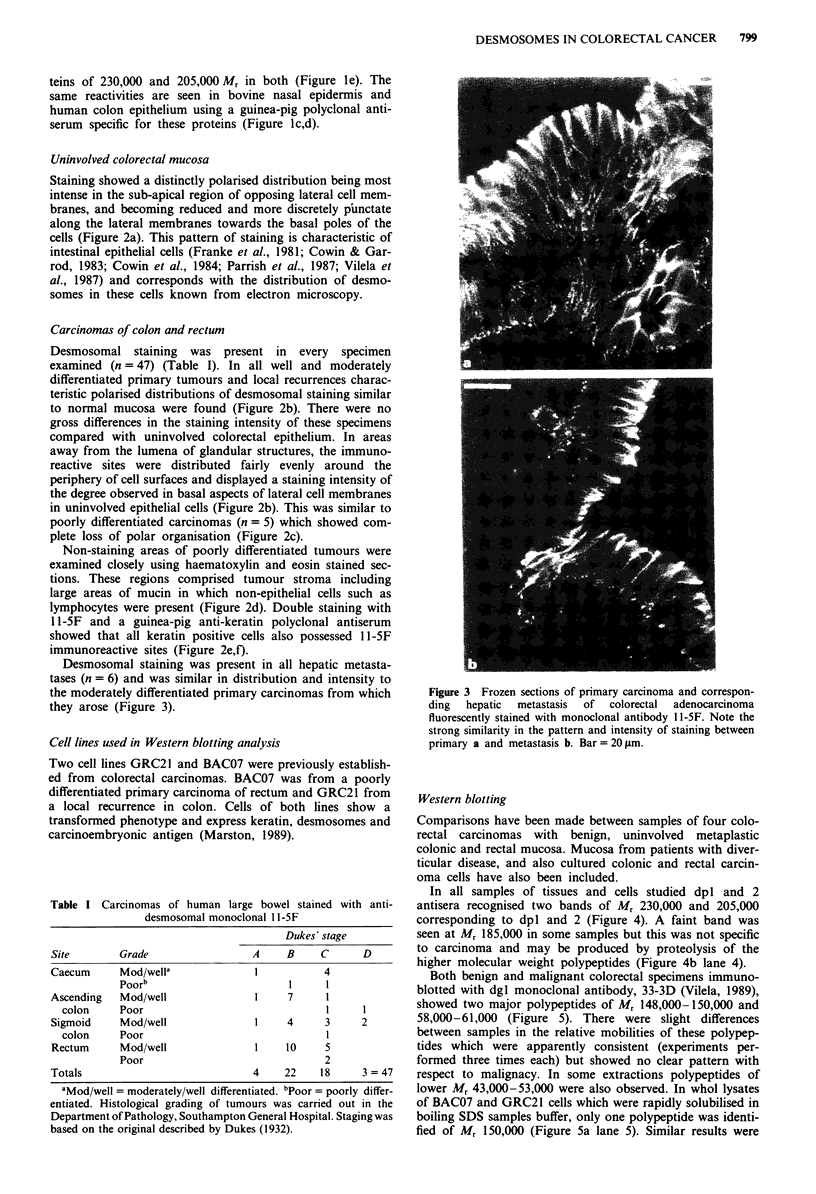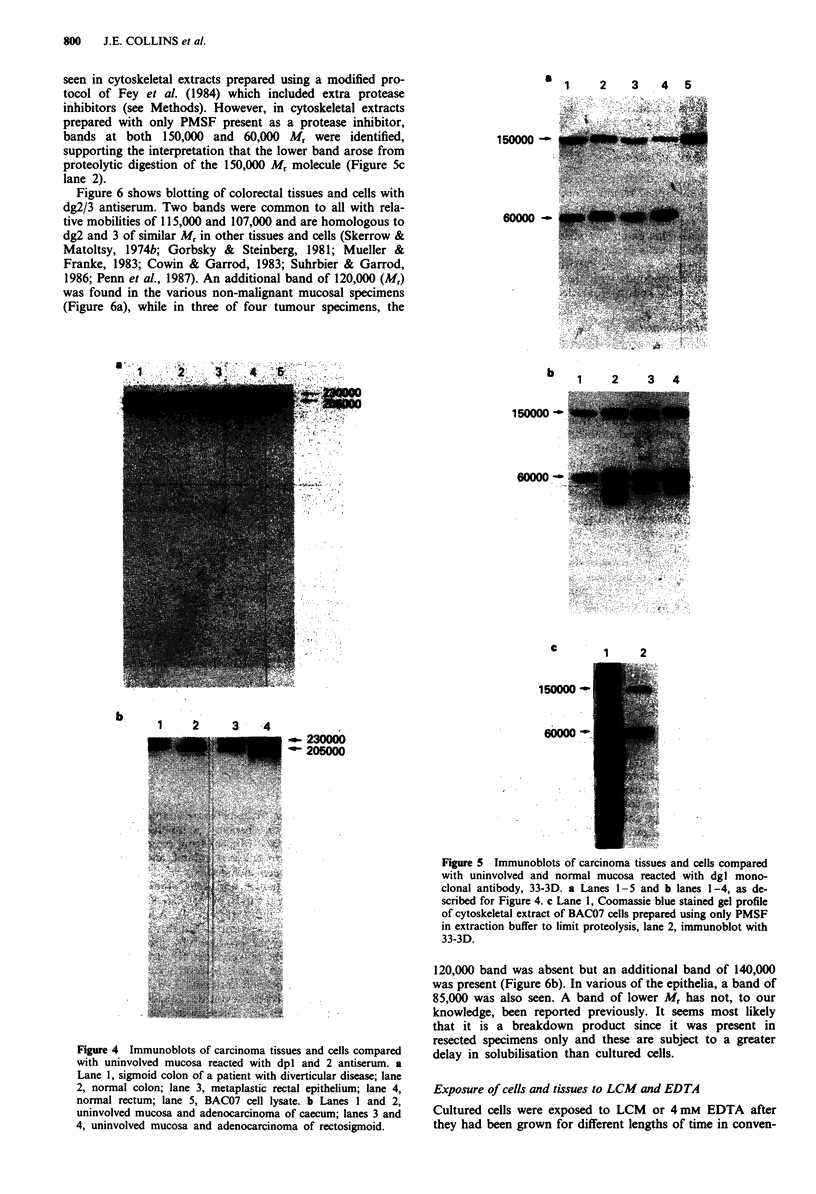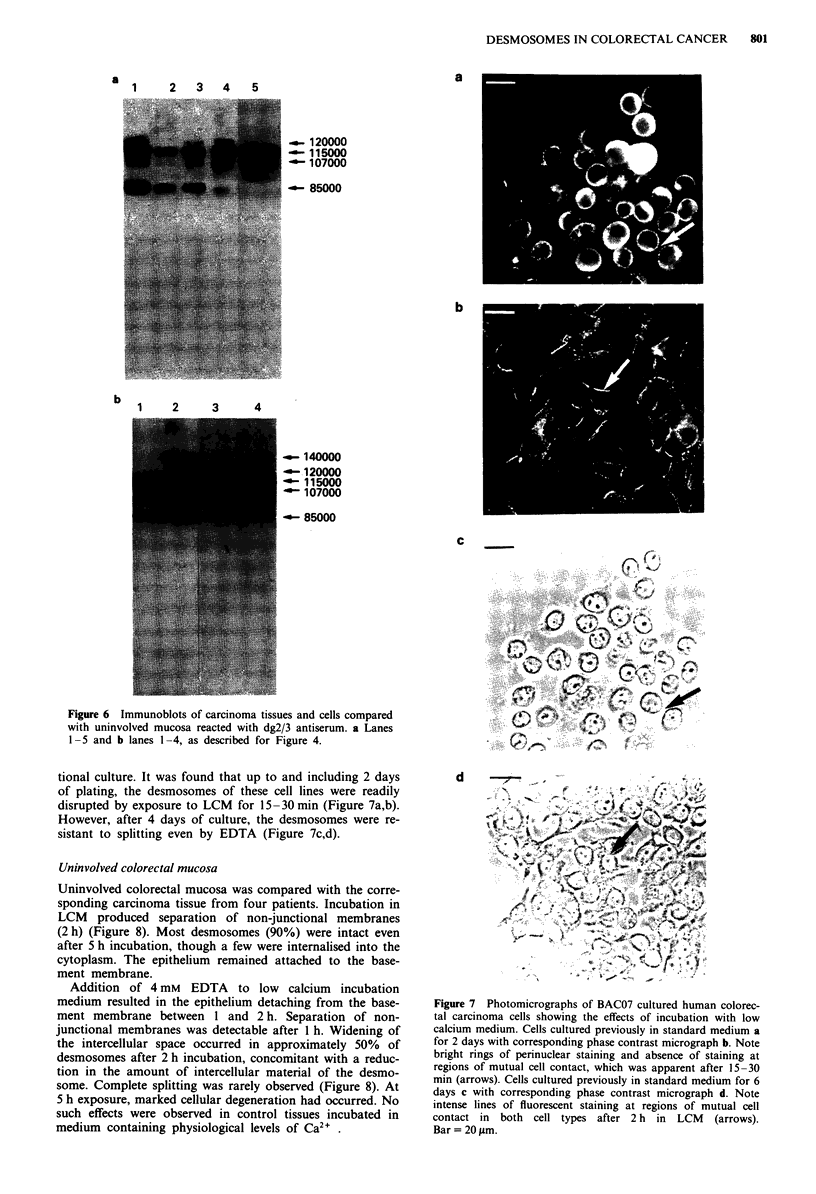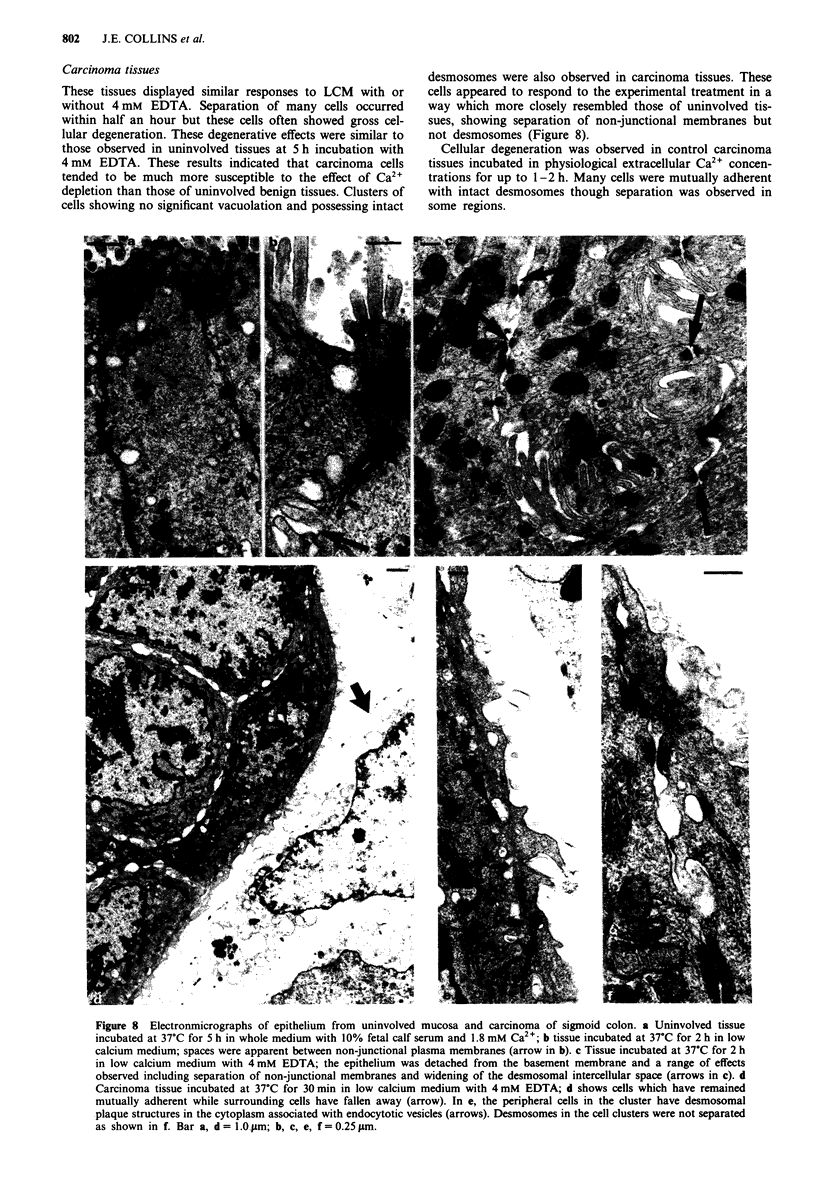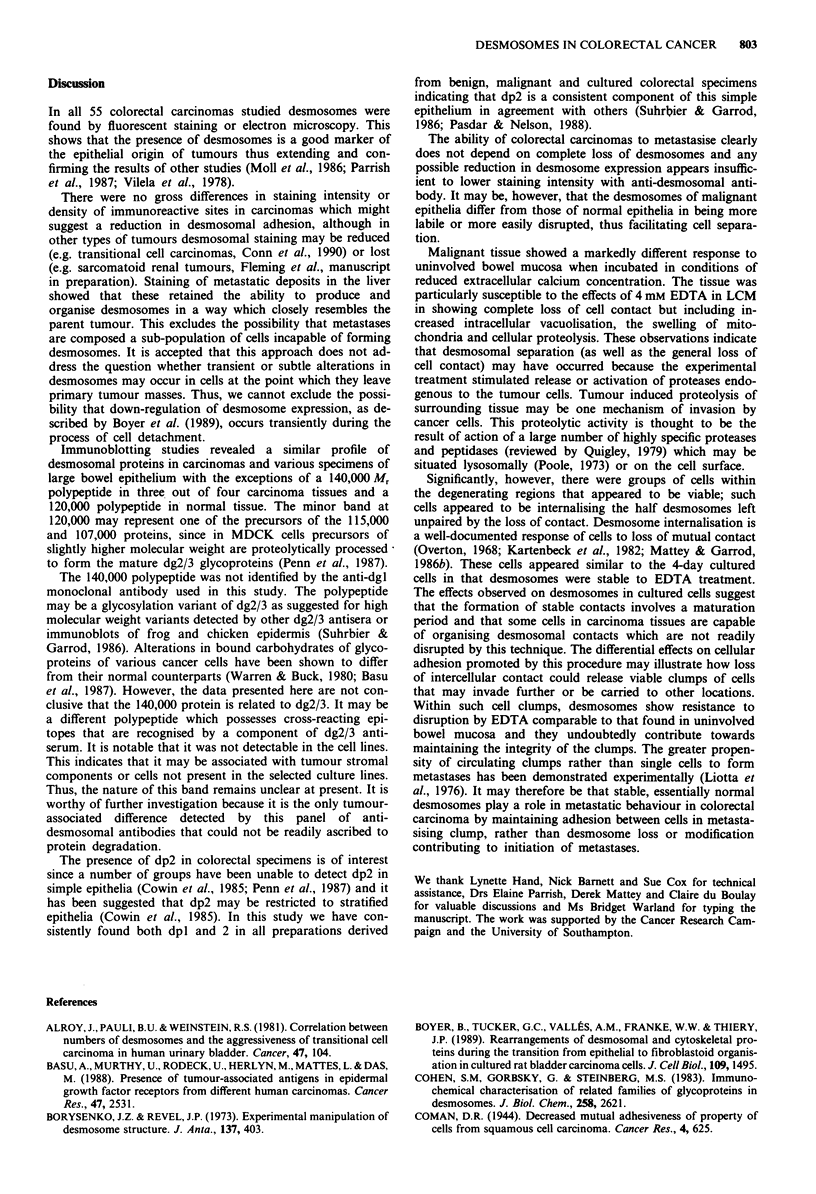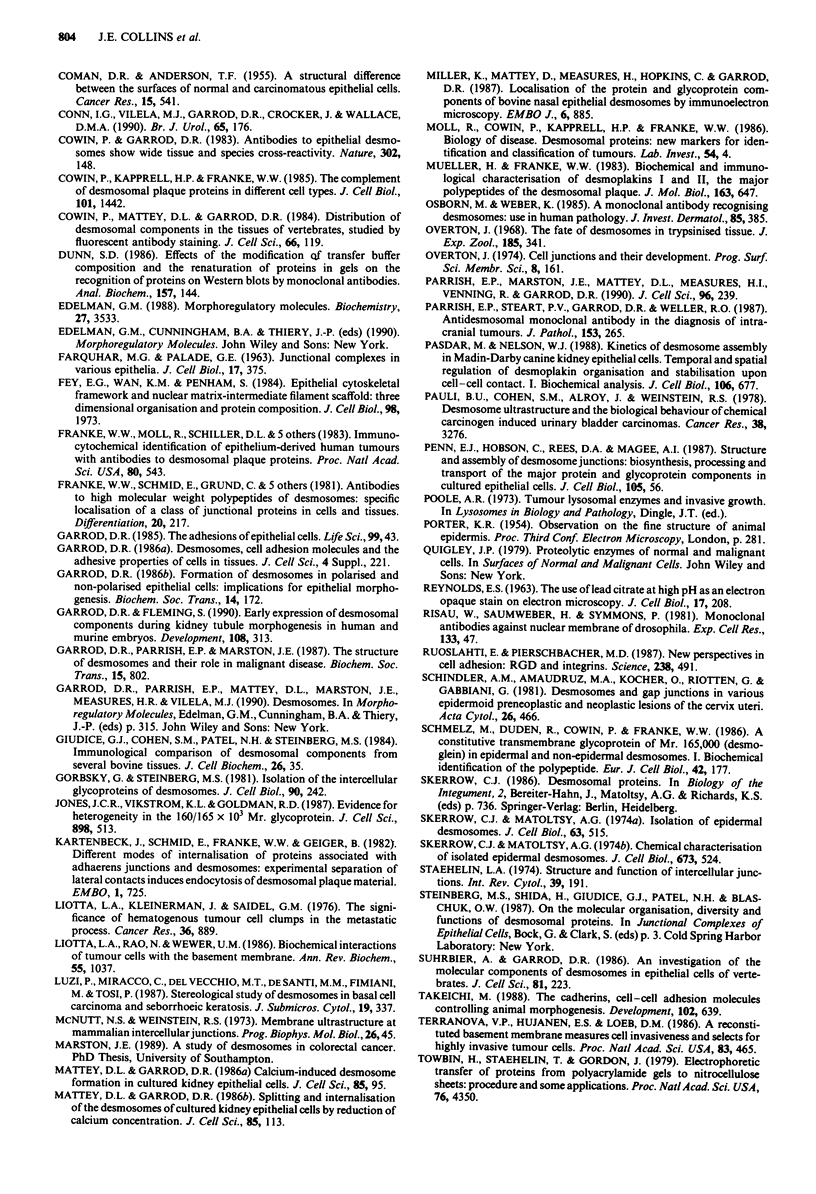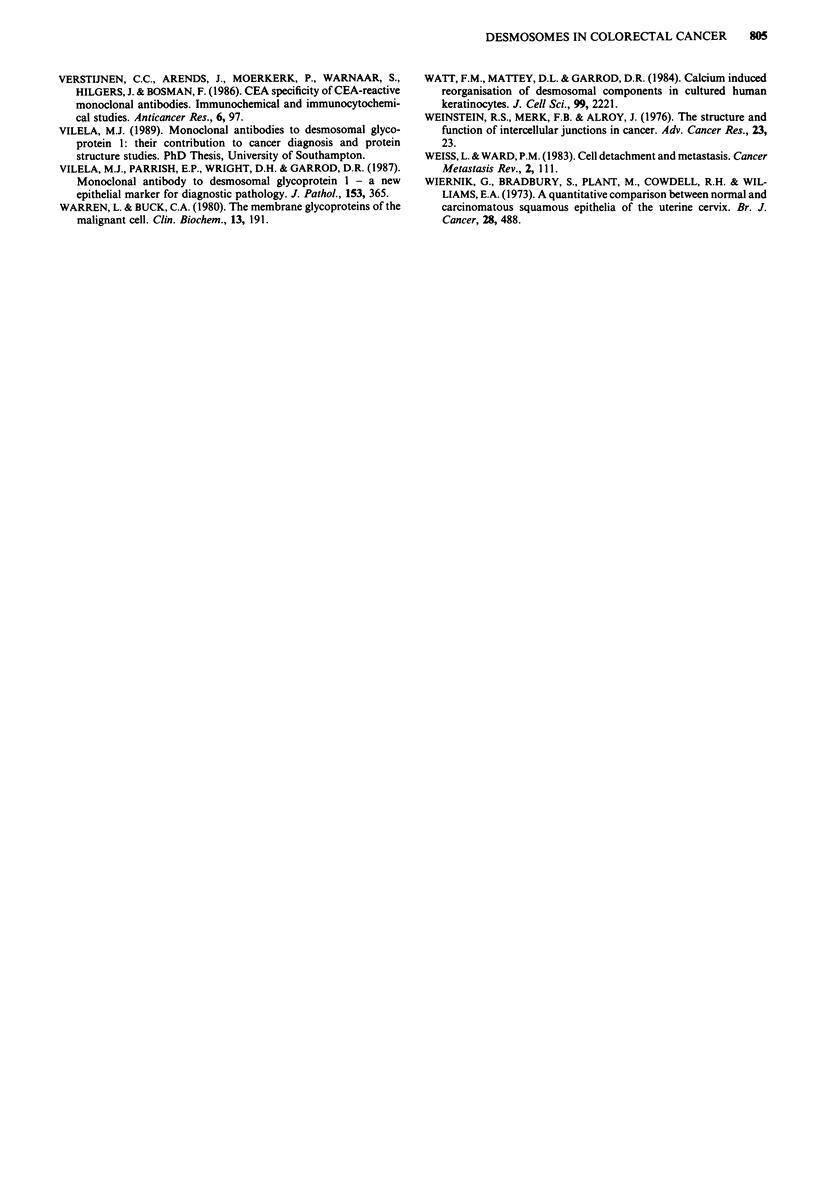# A study of desmosomes in colorectal carcinoma.

**DOI:** 10.1038/bjc.1990.382

**Published:** 1990-11

**Authors:** J. E. Collins, I. Taylor, D. R. Garrod

**Affiliations:** University Surgical Unit, University of Southampton, UK.

## Abstract

**Images:**


Br. J. Cancer (1990), 62, 796-805 © Macmillan Press Ltd., 1990

### A study of desmosomes in colorectal carcinoma

J.E. Collins[1,2], I. Taylor[1] & D.R. Garrod[2]

[1]*University Surgical Unit and* [2]*Cancer Research Campaign Medical Oncology Unit, University of Southampton, Southampton General Hospital, Southampton SO9 4XY, UK.*

**Summary** Desmosomes are adhesive junctions of epithelial cells. Their expression may be altered or lost in carcinomas resulting in reduced cellular adhesiveness. The desmosomes of colorectal carcinomas have been studied by fluorescent antibody staining, immunoblotting and electromicroscopy. A series of 58 malignant specimens, comprised of primary tumours and metastases, were desmosome positive. There was no indication of a comparative reduction in desmosome expression that might give rise to reduced adhesiveness of tumour cells, although loss of polarised junctional distribution in poorly differentiated tumours might have such a consequence. Western blotting analysis of colorectal cancers and cultured carcinoma cells identified desmosomal polypeptides dp1 + 2, dg1 and dg2 + 3 with similar relative molecular weights to normal homologues. In addition, a polypeptide of 140,000 was recognised only in malignant epithelium by anti-dg2 + 3 antiserum. The significance of this polypeptide is not understood. Tumours and uninvolved epithelium were exposed to low extracellular [$Ca^{2+}$] to test whether tumour desmosomes were of reduced stability. This caused much cellular degradation in tumours but some viable cell clumps possessed desmosomes resistant to disruption by low [$Ca^{2+}$]. Desmosomes may thus have a positive role in metastasis by maintaining intercellular adhesion between metastasising cells.

Detachment of cells from the primary tumour is an essential step in metastatic spread of malignant tumours. Coman (1944, 1955) and his colleagues showed that the cells of certain types of carcinomas may be more readily detached from each other than cells of normal tissues. It was suggested that defective adhesiveness of the carcinoma cells might be responsible for facilitating their detachment and, although several other causative factors (e.g. tumour necrosis, extracellular enzymes) may be involved (discussed by Weiss & Ward, 1983), it remains possible that altered adhesiveness of malignant cells may make an important contribution to initiation of both invasive and metastatic spread.

Recent advances in our understanding of the molecular nature of cell adhesion mechanisms (Edelman *et al.*, 1990) have provided a basis for the detailed analysis of cellular adhesiveness in neoplastic cells. The 'adhesiveness' of a cell is made up of the combined contributions from a number of different cell–cell and cell–substratum adhesion mechanisms, (Garrod, 1985, 1986*a*,*b*; Edelman, 1988; Takeichi, 1988; Ruoshlati & Pierschbacher, 1988). In this paper we shall be concerned with one of the intercellular junctional adhesion mechanisms of epithelial cells, the desmosome or *macula adhaerens*, and with one class of tumour, colorectal carcinoma.

There have been many studies of desmosomes in tumours by electron microscopy. Certain morphometric analyses have concluded that a reduction in desmosomal number correlates with invasive behaviour (Alroy *et al.*, 1981; Pauli *et al.*, 1978; Schindler *et al.*, 1981) while others have not substantiated this interpretation (Luzi *et al.*, 1987; Wiernik *et al.*, 1973). However, electron microscopical studies are necessarily based on small samples of tissue from each tumour and relatively small numbers of tumours. Such factors may contribute to these apparent contradictions. The use of antibody staining in studying larger areas of tumours with greater speed allows a more accurate impression of desmosomal density and distribution than electron microscopy (Franke *et al.*, 1983).

The major proteins and glycoproteins of desmosomes have been used for the production of specific antibodies (Franke *et al.*, 1981; Cowin & Garrod, 1983; Cohen *et al.*, 1983), some of which have been used in analysis of human cancer (Franke *et al.*, 1983; Osborn & Weber, 1985; Moll *et al.*, 1986; Garrod *et al.*, 1990; Parrish *et al.*, 1987; Vilela *et al.*, 1987). In this study, our first objective has been to use such antibodies to conduct a survey of a large number of specimens of human colorectal carcinoma by fluorescent antibody staining of frozen sections. In carrying out this study we have asked the following questions. (1) Do all specimens of colorectal carcinoma possess desmosomal staining? (2) Are there differences in the pattern of staining between normal or uninvolved bowel and primary and/or secondary carcinomas? (3) Are there gross and obvious differences between the amount of staining in tumours compared with normal or uninvolved bowel? Question 1 relates also to the possible use of desmosomal antigens as epithelial markers in tumour diagnosis. Clearly this depends upon knowing what proportion of tumours of a particular type are likely to possess the marker. In addition, we have used anti-desmosomal antibodies on Western blots to compare the relative electrophoretic mobilities of desmosomal antigens in tumours, tumour derived cultured cells, uninvolved and normal bowel.

Tumour desmosomes may be less stable than those in normal tissue and thus the adhesion between the tumour cells may be more labile. The desmosomes of certain normal epithelia, cultured human keratinocytes and MDCK cells show variable resistance to disruption by EDTA or reduced extracellular calcium concentrations [$Ca^{2+}$]. It has been speculated that this may reflect differences in stability (Borysenko & Revel, 1973; Watt *et al.*, 1984; Mattey & Garrod, 1986*b*). Recent evidence suggests lability of desmosomes in developing kidney tubule epithelium (Garrod & Fleming, 1990). In an attempt to compare the stability of desmosomes in uninvolved and malignant bowel epithelium to treatment with EDTA or reduced extracellular [$Ca^{2+}$] we have used electron microscopy to observe changes in desmosomal structure.

### Materials and methods

### *Tissues and tumours*

All tumours and uninvolved or normal tissues used in this study were obtained immediately after surgical resection. Staging of local and distant tumour spread was classified by a modification of Dukes (1932) and the degree of histological differentiation, as defined by the presence or absence of glandular ducts (Dukes & Bussey, 1958), was performed by the pathologists of Southampton General Hospital.

### *Antibodies*

Antisera and monoclonal antibodies were raised against protein or glycoprotein components of bovine nasal epithelial

Correspondence: J.E. Collins.
Received 5 March 1990; and in revised form 18 May 1990.

desmosomes purified by polyacrylamide gel electrophoresis as previously described (Cowin & Garrod, 1983; Parrish *et al.*, 1987). Antibody specificities determined by immunoblotting against whole bovine desmosomes are shown in Figure 1.

*Fluorescent antibody staining*

Tissues were snap frozen in liquid nitrogen. Sections of 6 $\mu$m were cut on a Slee cryostat, air dried for 90 min and stored, desiccated, at $-20$°C for up to 2 weeks. Before staining, sections were fixed in dehydrated acetone for 15 min at room temperature. This method was also used for staining of cells but, alternatively, cells were sometimes fixed in absolute methanol at 4°C for 10 min.

All antisera and antibodies were titrated on sample sections of colorectal mucosa or tumour in order to ascertain optimal working dilutions. Dilutions were carried out in Tris buffered saline (TBS) containing 0.1% sodium azide and 0.05% gelatine. To block non-specific binding of antibody or fluorescent conjugate the sections were incubated for 30 min at room temperature in Dulbecco's Modified Eagles Medium (DMEM) plus 1% bovine serum albumin (BSA) and 10% fetal calf serum (FCS). Diluted monoclonal antibody culture supernatant or polyclonal antisera were applied for 45 min at room temperature. After three 5-min washes in TBS affinity-purified fluorescent conjugate was applied for 30 min. The fluorescent conjugates used were sheep anti-mouse Ig (Amersham International) with monoclonal antibodies and rabbit anti-guinea pig Ig (Sigma Chemical Co.) with polyclonal antisera. After three 5-min washes in TBS the sections were mounted in glycerol:TBS (1:9, v:v) containing 25 mg ml$^{-1}$ 1,4-diazobicylco-(2,2,2)-octane (DABCO) an anti-bleaching agent (Johnson, 1982). Sections were examined by epi-illumination on a Zeiss Photomicroscope III.

*Polyacrylamide gel electrophoresis*

High density cultures of cells (1 week after plating) in tissue culture dishes were solubilised directly in boiling sample buffer. The sample was then boiled for 2 min, spun in a Beckman microfuge for 10 min and the supernatant stored at $-70$°C until required for electrophoresis.

For normal and malignant colorectal specimens the bowel was opened along the anti-mesenteric border immediately after removal from the patient. A piece of mucosa (4 × 2 cm approx.) was carefully removed from the submucosa with small scissors, avoiding the 2 cm perimeter near the resection margins. A wedge of tumour (1 cm$^3$ approx.) was taken. This included exophytic tumour and material from the deeper aspects. An adjacent piece of tumour was frozen in liquid nitrogen so that frozen sections of the specimen could be examined later to confirm malignancy.

**Figure 1** Western blot showing the specificities of desmosomal antibodies against bovine epidermal desmosomes and human colonic mucosa. Bovine epidermal desmosomes **a**, **b**, **c** and human colonic mucosa **d**, **e** reacted with guinea-pig, anti-dg2 + 3 serum **a**; 33-3D, dg1 monoclonal antibody **b**; guinea-pig, anti-dp1 + 2 serum **c**, **d**; 11-5F, dp1 + 2 monoclonal antibody **e**.

The samples were washed for 5 min in two changes of PBS containing 2.0 mM $CaCl_2$, 0.8 mM $MgCl_2$ and 4 mM phenylmethanesulphonylfluoride (PMSF). The specimens were then placed in either DMEM (Gibco Ltd) containing 4 mg ml$^{-1}$ bovine testicular hyaluronidase, L-1-tosylamide-2-phenylethylchloromethyketone (TPCK) (100 $\mu$g ml$^{-1}$), soy bean trypsin inhibitor (100 $\mu$g ml$^{-1}$), pepstatin A (80 $\mu$M), leupeptin (80 $\mu$M) and PMSF (4 mM) (all from Sigma) for a total time of 15 min. The tumour tissue was minced finely with a scalpel blades and gently stirred. The normal mucosa was stirred for 10 min and the epithelium was scraped off with scalpel blade. (Small samples of the scraped mucosa were fixed in 5% glutaraldehyde in 0.1 M cacodylate buffer in order to follow the processing by electron microscopy.) The mucosal scrapings or minced tumour were homogenised in an ultraturrax (IKA-WERK) and then centrifuged at 2,500 *g* for 15 min. The pellets were solubilised directly in boiling sample buffer using a vortex mixer to aid dispersal and boiled for a further 5 min. The samples were spun in a Beckman microfuge for 5 min and the supernatants stored in 100 $\mu$l aliquots at $-70$°C until required for electrophoresis. Samples were electrophoresed on SDS PAGE gels (Laemmli, 1970), in adjacent lanes to enable direct comparisons of mobilities.

*Transfer of proteins onto nitrocellulose membranes*

After electrophoresis gels were soaked in transfer buffer (Towbin *et al.*, 1979) for 20 min with or without prior renaturation in urea buffer (Risau *et al.*, 1981). Proteins were tranferred to nitrocellulose membranes (Amersham International plc) in a Tris/glycine buffer pH 8.3 (Towbin *et al.*, 1979) or in carbonate/bicarbonate buffer pH 9.9 (Dunn, 1986). After transfer the membranes were dried overnight at room temperature and stored desiccated at 4°C in airtight plastic bags until required.

Immunolocalisation of specific proteins bound to nitrocellulose membranes was essentially as described by Suhrbier & Garrod (1986) for polyclonal antibodies. For monoclonal antibodies the high salt washes described by Suhrbier & Garrod were omitted and $^{125}$I-labelled affinity-purified sheep anti-mouse Ig used instead of $^{125}$I-protein A to detect the antibody binding. Controls included DMEM instead of specific antibody supernatants for monoclonals and non-immune or pre-injection serum instead of guinea-pig antibodies.

*Exposure of tissues to reduced extracellular divalent cation concentration*

Uninvolved colorectal mucosa and tumour tissue blocks (1 mm$^3$) were incubated in 10 ml of medium (per five blocks) of low calcium medium (LCM) or LCM with 4 mM EDTA (pH 7.4). LCM consisted of a 3:1 mixture of DMEM and Ham's F12 medium without calcium salts (Imperial Laboratories, Salisbury) containing 10% FCS that had been depleted of divalent cations with Chelex 100 resin (BioRad). The $Ca^{2+}$ concentration of this medium was 0.04–0.05 mM as determined by atomic absorption spectrophotometry. Control tissues were incubated in similar medium containing approx. 1.8 mM $Ca^{2+}$ and without EDTA. Incubations were carried out at 37°C on a Denby Spiramix for varying time intervals.

*Electron microscopy*

Tissue blocks were fixed in 5% glutaraldehyde in 100 mM sodium cacodylate at pH 7.4 containing 2 mM $CaCl_2$ for 8 h at room temperature. Fixative was replaced by 250 mM sucrose in cacodylate buffer for 24 h at 4°C. Post-fixation was with 2% osmium tetroxide in cacodylate buffer for 2 h at room temperature followed by *en bloc* uranyl acetate staining. Dehydration through an ethanol series was followed by embedding in Spurr resin. Sections were cut on a Reichert MT-2B ultramicrotome. Thick sections (~0.5 μm) were stained with toluidine blue. Thin sections (~0.07 μm) were stained with a filtered lead citrate (Reynolds, 1963) and examined on a Phillips 201 electron microscope.

### Results

### *Fluorescent antibody staining*

The staining of desmosomes was performed with monoclonal antibody, 11-5F, to dp1 and 2 (Parrish *et al.*, 1987). Western blots showing the specificity of monoclonal antibody, 11-5F, on human colonic epithelium show reaction with two pro-

**Figure 2** Fluorescent staining of frozen sections of human colonic mucosa and primary carcinomas. In normal mucosa **a** staining was specific for the epithelium with enrichment at the junctional complexes of the cells (arrowhead), (l = lumen, p = lamina propria, e = epithelium). In moderately-differentiated primary carcinomas (**b**, Dukes C, caecum) polarised distributions of staining were seen (arrowhead) with similar staining intensity to normal. Poorly-differentiated carcinoma (**c**, Dukes C, lower rectum) showed loss of polarised staining. 11-5F non-staining areas examined on haematoxylin and eosin stained sections **d** corresponded to mucin and tumour stroma (s = stroma). Monoclonal 11-5F **e** and affinity-purified guinea pig anti-cytokeratin **f** staining of Dukes C carcinoma from lower rectum. Corresponding staining areas co-express desmosomal and cytokeratin antigens. Bar = 20 μm.

teins of 230,000 and 205,000 $M_r$ in both (Figure 1e). The same reactivities are seen in bovine nasal epidermis and human colon epithelium using a guinea-pig polyclonal antiserum specific for these proteins (Figure 1c,d).

*Uninvolved colorectal mucosa*

Staining showed a distinctly polarised distribution being most intense in the sub-apical region of opposing lateral cell membranes, and becoming reduced and more discretely punctate along the lateral membranes towards the basal poles of the cells (Figure 2a). This pattern of staining is characteristic of intestinal epithelial cells (Franke *et al.*, 1981; Cowin & Garrod, 1983; Cowin *et al.*, 1984; Parrish *et al.*, 1987; Vilela *et al.*, 1987) and corresponds with the distribution of desmosomes in these cells known from electron microscopy.

*Carcinomas of colon and rectum*

Desmosomal staining was present in every specimen examined ($n = 47$) (Table I). In all well and moderately differentiated primary tumours and local recurrences characteristic polarised distributions of desmosomal staining similar to normal mucosa were found (Figure 2b). There were no gross differences in the staining intensity of these specimens compared with uninvolved colorectal epithelium. In areas away from the lumena of glandular structures, the immunoreactive sites were distributed fairly evenly around the periphery of cell surfaces and displayed a staining intensity of the degree observed in basal aspects of lateral cell membranes in uninvolved epithelial cells (Figure 2b). This was similar to poorly differentiated carcinomas ($n = 5$) which showed complete loss of polar organisation (Figure 2c).

Non-staining areas of poorly differentiated tumours were examined closely using haematoxylin and eosin stained sections. These regions comprised tumour stroma including large areas of mucin in which non-epithelial cells such as lymphocytes were present (Figure 2d). Double staining with 11-5F and a guinea-pig anti-keratin polyclonal antiserum showed that all keratin positive cells also possessed 11-5F immunoreactive sites (Figure 2e,f).

Desmosomal staining was present in all hepatic metastatases ($n = 6$) and was similar in distribution and intensity to the moderately differentiated primary carcinomas from which they arose (Figure 3).

*Cell lines used in Western blotting analysis*

Two cell lines GRC21 and BAC07 were previously established from colorectal carcinomas. BAC07 was from a poorly differentiated primary carcinoma of rectum and GRC21 from a local recurrence in colon. Cells of both lines show a transformed phenotype and express keratin, desmosomes and carcinoembryonic antigen (Marston, 1989).

**Table I** Carcinomas of human large bowel stained with anti-desmosomal monoclonal 11-5F

| | | *Dukes' stage* | | | |
|---|---|---|---|---|---|
| *Site* | *Grade* | *A* | *B* | *C* | *D* |
| Caecum | Mod/well[a] | 1 | | 4 | |
| | Poor[b] | | 1 | 1 | |
| Ascending | Mod/well | 1 | 7 | 1 | |
| colon | Poor | | | 1 | 1 |
| Sigmoid | Mod/well | 1 | 4 | 3 | 2 |
| colon | Poor | | | 1 | |
| Rectum | Mod/well | 1 | 10 | 5 | |
| | Poor | | | 2 | |
| Totals | | 4 | 22 | 18 | 3 = 47 |

[a]Mod/well = moderately/well differentiated. [b]Poor = poorly differentiated. Histological grading of tumours was carried out in the Department of Pathology, Southampton General Hospital. Staging was based on the original described by Dukes (1932).

**Figure 3** Frozen sections of primary carcinoma and corresponding hepatic metastasis of colorectal adenocarcinoma fluorescently stained with monoclonal antibody 11-5F. Note the strong similarity in the pattern and intensity of staining between primary **a** and metastasis **b**. Bar = 20 μm.

*Western blotting*

Comparisons have been made between samples of four colorectal carcinomas with benign, uninvolved metaplastic colonic and rectal mucosa. Mucosa from patients with diverticular disease, and also cultured colonic and rectal carcinoma cells have also been included.

In all samples of tissues and cells studied dpl and 2 antisera recognised two bands of $M_r$ 230,000 and 205,000 corresponding to dpl and 2 (Figure 4). A faint band was seen at $M_r$ 185,000 in some samples but this was not specific to carcinoma and may be produced by proteolysis of the higher molecular weight polypeptides (Figure 4b lane 4).

Both benign and malignant colorectal specimens immunoblotted with dgl monoclonal antibody, 33-3D (Vilela, 1989), showed two major polypeptides of $M_r$ 148,000–150,000 and 58,000–61,000 (Figure 5). There were slight differences between samples in the relative mobilities of these polypeptides which were apparently consistent (experiments performed three times each) but showed no clear pattern with respect to malignancy. In some extractions polypeptides of lower $M_r$ 43,000–53,000 were also observed. In whol lysates of BAC07 and GRC21 cells which were rapidly solubilised in boiling SDS samples buffer, only one polypeptide was identified of $M_r$ 150,000 (Figure 5a lane 5). Similar results were seen in cytoskeletal extracts prepared using a modified protocol of Fey *et al.* (1984) which included extra protease inhibitors (see Methods). However, in cytoskeletal extracts prepared with only PMSF present as a protease inhibitor, bands at both 150,000 and 60,000 $M_r$ were identified, supporting the interpretation that the lower band arose from proteolytic digestion of the 150,000 $M_r$ molecule (Figure 5c lane 2).

Figure 6 shows blotting of colorectal tissues and cells with dg2/3 antiserum. Two bands were common to all with relative mobilities of 115,000 and 107,000 and are homologous to dg2 and 3 of similar $M_r$ in other tissues and cells (Skerrow & Matoltsy, 1974*b*; Gorbsky & Steinberg, 1981; Mueller & Franke, 1983; Cowin & Garrod, 1983; Suhrbier & Garrod, 1986; Penn *et al.*, 1987). An additional band of 120,000 ($M_r$) was found in the various non-malignant mucosal specimens (Figure 6a), while in three of four tumour specimens, the 120,000 band was absent but an additional band of 140,000 was present (Figure 6b). In various of the epithelia, a band of 85,000 was also seen. A band of lower $M_r$ has not, to our knowledge, been reported previously. It seems most likely that it is a breakdown product since it was present in resected specimens only and these are subject to a greater delay in solubilisation than cultured cells.

**Figure 4** Immunoblots of carcinoma tissues and cells compared with uninvolved mucosa reacted with dp1 and 2 antiserum. **a** Lane 1, sigmoid colon of a patient with diverticular disease; lane 2, normal colon; lane 3, metaplastic rectal epithelium; lane 4, normal rectum; lane 5, BAC07 cell lysate. **b** Lanes 1 and 2, uninvolved mucosa and adenocarcinoma of caecum; lanes 3 and 4, uninvolved mucosa and adenocarcinoma of rectosigmoid.

**Figure 5** Immunoblots of carcinoma tissues and cells compared with uninvolved and normal mucosa reacted with dg1 monoclonal antibody, 33-3D. **a** Lanes 1–5 and **b** lanes 1–4, as described for Figure 4. **c** Lane 1, Coomassie blue stained gel profile of cytoskeletal extract of BAC07 cells prepared using only PMSF in extraction buffer to limit proteolysis, lane 2, immunoblot with 33-3D.

### *Exposure of cells and tissues to LCM and EDTA*

Cultured cells were exposed to LCM or 4 mM EDTA after they had been grown for different lengths of time in conven-

**Figure 6** Immunoblots of carcinoma tissues and cells compared with uninvolved mucosa reacted with dg2/3 antiserum. **a** Lanes 1–5 and **b** lanes 1–4, as described for Figure 4.

tional culture. It was found that up to and including 2 days of plating, the desmosomes of these cell lines were readily disrupted by exposure to LCM for 15–30 min (Figure 7a,b). However, after 4 days of culture, the desmosomes were resistant to splitting even by EDTA (Figure 7c,d).

*Uninvolved colorectal mucosa*

Uninvolved colorectal mucosa was compared with the corresponding carcinoma tissue from four patients. Incubation in LCM produced separation of non-junctional membranes (2 h) (Figure 8). Most desmosomes (90%) were intact even after 5 h incubation, though a few were internalised into the cytoplasm. The epithelium remained attached to the basement membrane.

Addition of 4 mM EDTA to low calcium incubation medium resulted in the epithelium detaching from the basement membrane between 1 and 2 h. Separation of non-junctional membranes was detectable after 1 h. Widening of the intercellular space occurred in approximately 50% of desmosomes after 2 h incubation, concomitant with a reduction in the amount of intercellular material of the desmosome. Complete splitting was rarely observed (Figure 8). At 5 h exposure, marked cellular degeneration had occurred. No such effects were observed in control tissues incubated in medium containing physiological levels of $Ca^{2+}$ .

**Figure 7** Photomicrographs of BAC07 cultured human colorectal carcinoma cells showing the effects of incubation with low calcium medium. Cells cultured previously in standard medium **a** for 2 days with corresponding phase contrast micrograph **b**. Note bright rings of perinuclear staining and absence of staining at regions of mutual cell contact, which was apparent after 15–30 min (arrows). Cells cultured previously in standard medium for 6 days **c** with corresponding phase contrast micrograph **d**. Note intense lines of fluorescent staining at regions of mutual cell contact in both cell types after 2 h in LCM (arrows). Bar = 20 μm.

*Carcinoma tissues*

These tissues displayed similar responses to LCM with or without 4 mM EDTA. Separation of many cells occurred within half an hour but these cells often showed gross cellular degeneration. These degenerative effects were similar to those observed in uninvolved tissues at 5 h incubation with 4 mM EDTA. These results indicated that carcinoma cells tended to be much more susceptible to the effect of $Ca^{2+}$ depletion than those of uninvolved benign tissues. Clusters of cells showing no significant vacuolation and possessing intact desmosomes were also observed in carcinoma tissues. These cells appeared to respond to the experimental treatment in a way which more closely resembled those of uninvolved tissues, showing separation of non-junctional membranes but not desmosomes (Figure 8).

Cellular degeneration was observed in control carcinoma tissues incubated in physiological extracellular $Ca^{2+}$ concentrations for up to 1–2 h. Many cells were mutually adherent with intact desmosomes though separation was observed in some regions.

**Figure 8** Electronmicrographs of epithelium from uninvolved mucosa and carcinoma of sigmoid colon. **a** Uninvolved tissue incubated at 37°C for 5 h in whole medium with 10% fetal calf serum and 1.8 mM $Ca^{2+}$; **b** tissue incubated at 37°C for 2 h in low calcium medium; spaces were apparent between non-junctional plasma membranes (arrow in **b**). **c** Tissue incubated at 37°C for 2 h in low calcium medium with 4 mM EDTA; the epithelium was detached from the basement membrane and a range of effects observed including separation of non-junctional membranes and widening of the desmosomal intercellular space (arrows in **c**). **d** Carcinoma tissue incubated at 37°C for 30 min in low calcium medium with 4 mM EDTA; **d** shows cells which have remained mutually adherent while surrounding cells have fallen away (arrow). In **e**, the peripheral cells in the cluster have desmosomal plaque structures in the cytoplasm associated with endocytotic vesicles (arrows). Desmosomes in the cell clusters were not separated as shown in **f**. Bar **a**, **d** = 1.0 μm; **b**, **c**, **e**, **f** = 0.25 μm.

### Discussion

In all 55 colorectal carcinomas studied desmosomes were found by fluorescent staining or electron microscopy. This shows that the presence of desmosomes is a good marker of the epithelial origin of tumours thus extending and confirming the results of other studies (Moll *et al.*, 1986; Parrish *et al.*, 1987; Vilela *et al.*, 1978).

There were no gross differences in staining intensity or density of immunoreactive sites in carcinomas which might suggest a reduction in desmosomal adhesion, although in other types of tumours desmosomal staining may be reduced (e.g. transitional cell carcinomas, Conn *et al.*, 1990) or lost (e.g. sarcomatoid renal tumours, Fleming *et al.*, manuscript in preparation). Staining of metastatic deposits in the liver showed that these retained the ability to produce and organise desmosomes in a way which closely resembles the parent tumour. This excludes the possibility that metastases are composed a sub-population of cells incapable of forming desmosomes. It is accepted that this approach does not address the question whether transient or subtle alterations in desmosomes may occur in cells at the point which they leave primary tumour masses. Thus, we cannot exclude the possibility that down-regulation of desmosome expression, as described by Boyer *et al.* (1989), occurs transiently during the process of cell detachment.

Immunoblotting studies revealed a similar profile of desmosomal proteins in carcinomas and various specimens of large bowel epithelium with the exceptions of a 140,000 $M_r$ polypeptide in three out of four carcinoma tissues and a 120,000 polypeptide in normal tissue. The minor band at 120,000 may represent one of the precursors of the 115,000 and 107,000 proteins, since in MDCK cells precursors of slightly higher molecular weight are proteolytically processed to form the mature dg2/3 glycoproteins (Penn *et al.*, 1987).

The 140,000 polypeptide was not identified by the anti-dg1 monoclonal antibody used in this study. The polypeptide may be a glycosylation variant of dg2/3 as suggested for high molecular weight variants detected by other dg2/3 antisera or immunoblots of frog and chicken epidermis (Suhrbier & Garrod, 1986). Alterations in bound carbohydrates of glycoproteins of various cancer cells have been shown to differ from their normal counterparts (Warren & Buck, 1980; Basu *et al.*, 1987). However, the data presented here are not conclusive that the 140,000 protein is related to dg2/3. It may be a different polypeptide which possesses cross-reacting epitopes that are recognised by a component of dg2/3 antiserum. It is notable that it was not detectable in the cell lines. This indicates that it may be associated with tumour stromal components or cells not present in the selected culture lines. Thus, the nature of this band remains unclear at present. It is worthy of further investigation because it is the only tumour-associated difference detected by this panel of anti-desmosomal antibodies that could not be readily ascribed to protein degradation.

The presence of dp2 in colorectal specimens is of interest since a number of groups have been unable to detect dp2 in simple epithelia (Cowin *et al.*, 1985; Penn *et al.*, 1987) and it has been suggested that dp2 may be restricted to stratified epithelia (Cowin *et al.*, 1985). In this study we have consistently found both dp1 and 2 in all preparations derived from benign, malignant and cultured colorectal specimens indicating that dp2 is a consistent component of this simple epithelium in agreement with others (Suhrbier & Garrod, 1986; Pasdar & Nelson, 1988).

The ability of colorectal carcinomas to metastasise clearly does not depend on complete loss of desmosomes and any possible reduction in desmosome expression appears insufficient to lower staining intensity with anti-desmosomal antibody. It may be, however, that the desmosomes of malignant epithelia differ from those of normal epithelia in being more labile or more easily disrupted, thus facilitating cell separation.

Malignant tissue showed a markedly different response to uninvolved bowel mucosa when incubated in conditions of reduced extracellular calcium concentration. The tissue was particularly susceptible to the effects of 4 mM EDTA in LCM in showing complete loss of cell contact but including increased intracellular vacuolisation, the swelling of mitochondria and cellular proteolysis. These observations indicate that desmosomal separation (as well as the general loss of cell contact) may have occurred because the experimental treatment stimulated release or activation of proteases endogenous to the tumour cells. Tumour induced proteolysis of surrounding tissue may be one mechanism of invasion by cancer cells. This proteolytic activity is thought to be the result of action of a large number of highly specific proteases and peptidases (reviewed by Quigley, 1979) which may be situated lysosomally (Poole, 1973) or on the cell surface.

Significantly, however, there were groups of cells within the degenerating regions that appeared to be viable; such cells appeared to be internalising the half desmosomes left unpaired by the loss of contact. Desmosome internalisation is a well-documented response of cells to loss of mutual contact (Overton, 1968; Kartenbeck *et al.*, 1982; Mattey & Garrod, 1986*b*). These cells appeared similar to the 4-day cultured cells in that desmosomes were stable to EDTA treatment. The effects observed on desmosomes in cultured cells suggest that the formation of stable contacts involves a maturation period and that some cells in carcinoma tissues are capable of organising desmosomal contacts which are not readily disrupted by this technique. The differential effects on cellular adhesion promoted by this procedure may illustrate how loss of intercellular contact could release viable clumps of cells that may invade further or be carried to other locations. Within such cell clumps, desmosomes show resistance to disruption by EDTA comparable to that found in uninvolved bowel mucosa and they undoubtedly contribute towards maintaining the integrity of the clumps. The greater propensity of circulating clumps rather than single cells to form metastases has been demonstrated experimentally (Liotta *et al.*, 1976). It may therefore be that stable, essentially normal desmosomes play a role in metastatic behaviour in colorectal carcinoma by maintaining adhesion between cells in metastasising clump, rather than desmosome loss or modification contributing to initiation of metastases.

We thank Lynette Hand, Nick Barnett and Sue Cox for technical assistance, Drs Elaine Parrish, Derek Mattey and Claire du Boulay for valuable discussions and Ms Bridget Warland for typing the manuscript. The work was supported by the Cancer Research Campaign and the University of Southampton.

### References

ALROY, J., PAULI, B.U. & WEINSTEIN, R.S. (1981). Correlation between numbers of desmosomes and the aggressiveness of transitional cell carcinoma in human urinary bladder. *Cancer*, **47**, 104.

BASU, A., MURTHY, U., RODECK, U., HERLYN, M., MATTES, L. & DAS, M. (1988). Presence of tumour-associated antigens in epidermal growth factor receptors from different human carcinomas. *Cancer Res.*, **47**, 2531.

BORYSENKO, J.Z. & REVEL, J.P. (1973). Experimental manipulation of desmosome structure. *J. Anta.*, **137**, 403.

BOYER, B., TUCKER, G.C., VALLÉS, A.M., FRANKE, W.W. & THIERY, J.P. (1989). Rearrangements of desmosomal and cytoskeletal proteins during the transition from epithelial to fibroblastoid organisation in cultured rat bladder carcinoma cells. *J. Cell Biol.*, **109**, 1495.

COHEN, S.M, GORBSKY, G. & STEINBERG, M.S. (1983). Immunochemical characterisation of related families of glycoproteins in desmosomes. *J. Biol. Chem.*, **258**, 2621.

COMAN, D.R. (1944). Decreased mutual adhesiveness of property of cells from squamous cell carcinoma. *Cancer Res.*, **4**, 625.

COMAN, D.R. & ANDERSON, T.F. (1955). A structural difference between the surfaces of normal and carcinomatous epithelial cells. *Cancer Res.*, **15**, 541.

CONN, I.G., VILELA, M.J., GARROD, D.R., CROCKER, J. & WALLACE, D.M.A. (1990). *Br. J. Urol.*, **65**, 176.

COWIN, P. & GARROD, D.R. (1983). Antibodies to epithelial desmosomes show wide tissue and species cross-reactivity. *Nature*, **302**, 148.

COWIN, P., KAPPRELL, H.P. & FRANKE, W.W. (1985). The complement of desmosomal plaque proteins in different cell types. *J. Cell Biol.*, **101**, 1442.

COWIN, P., MATTEY, D.L. & GARROD, D.R. (1984). Distribution of desmosomal components in the tissues of vertebrates, studied by fluorescent antibody staining. *J. Cell Sci.*, **66**, 119.

DUNN, S.D. (1986). Effects of the modification of transfer buffer composition and the renaturation of proteins in gels on the recognition of proteins on Western blots by monoclonal antibodies. *Anal. Biochem.*, **157**, 144.

EDELMAN, G.M. (1988). Morphoregulatory molecules. *Biochemistry*, **27**, 3533.

EDELMAN, G.M., CUNNINGHAM, B.A. & THIERY, J.-P. (eds) (1990). *Morphoregulatory Molecules*. John Wiley and Sons: New York.

FARQUHAR, M.G. & PALADE, G.E. (1963). Junctional complexes in various epithelia. *J. Cell Biol.*, **17**, 375.

FEY, E.G., WAN, K.M. & PENHAM, S. (1984). Epithelial cytoskeletal framework and nuclear matrix-intermediate filament scaffold: three dimensional organisation and protein composition. *J. Cell Biol.*, **98**, 1973.

FRANKE, W.W., MOLL, R., SCHILLER, D.L. & 5 others (1983). Immunocytochemical identification of epithelium-derived human tumours with antibodies to desmosomal plaque proteins. *Proc. Natl Acad. Sci. USA*, **80**, 543.

FRANKE, W.W., SCHMID, E., GRUND, C. & 5 others (1981). Antibodies to high molecular weight polypeptides of desmosomes: specific localisation of a class of junctional proteins in cells and tissues. *Differentiation*, **20**, 217.

GARROD, D.R. (1985). The adhesions of epithelial cells. *Life Sci.*, **99**, 43.

GARROD, D.R. (1986*a*). Desmosomes, cell adhesion molecules and the adhesive properties of cells in tissues. *J. Cell Sci.*, 4 Suppl., 221.

GARROD, D.R. (1986*b*). Formation of desmosomes in polarised and non-polarised epithelial cells: implications for epithelial morphogenesis. *Biochem. Soc. Trans.*, **14**, 172.

GARROD, D.R. & FLEMING, S. (1990). Early expression of desmosomal components during kidney tubule morphogenesis in human and murine embryos. *Development*, **108**, 313.

GARROD, D.R., PARRISH, E.P. & MARSTON, J.E. (1987). The structure of desmosomes and their role in malignant disease. *Biochem. Soc. Trans.*, **15**, 802.

GARROD, D.R., PARRISH, E.P., MATTEY, D.L., MARSTON, J.E., MEASURES, H.R. & VILELA, M.J. (1990). Desmosomes. In *Morphoregulatory Molecules*, Edelman, G.M., Cunningham, B.A. & Thiery, J.-P. (eds) p. 315. John Wiley and Sons: New York.

GIUDICE, G.J., COHEN, S.M., PATEL, N.H. & STEINBERG, M.S. (1984). Immunological comparison of desmosomal components from several bovine tissues. *J. Cell Biochem.*, **26**, 35.

GORBSKY, G. & STEINBERG, M.S. (1981). Isolation of the intercellular glycoproteins of desmosomes. *J. Cell Biol.*, **90**, 242.

JONES, J.C.R., VIKSTROM, K.L. & GOLDMAN, R.D. (1987). Evidence for heterogeneity in the $160/165 \times 10^3$ Mr. glycoprotein. *J. Cell Sci.*, **898**, 513.

KARTENBECK, J., SCHMID, E., FRANKE, W.W. & GEIGER, B. (1982). Different modes of internalisation of proteins associated with adhaerens junctions and desmosomes: experimental separation of lateral contacts induces endocytosis of desmosomal plaque material. *EMBO*, **1**, 725.

LIOTTA, L.A., KLEINERMAN, J. & SAIDEL, G.M. (1976). The significance of hematogenous tumour cell clumps in the metastatic process. *Cancer Res.*, **36**, 889.

LIOTTA, L.A., RAO, N. & WEWER, U.M. (1986). Biochemical interactions of tumour cells with the basement membrane. *Ann. Rev. Biochem.*, **55**, 1037.

LUZI, P., MIRACCO, C., DEL VECCHIO, M.T., DE SANTI, M.M., FIMIANI, M. & TOSI, P. (1987). Stereological study of desmosomes in basal cell carcinoma and seborrhoeic keratosis. *J. Submicros. Cytol.*, **19**, 337.

MCNUTT, N.S. & WEINSTEIN, R.S. (1973). Membrane ultrastructure at mammalian intercellular junctions. *Prog. Biophys. Mol. Biol.*, **26**, 45.

MARSTON, J.E. (1989). A study of desmosomes in colorectal cancer. PhD Thesis, University of Southampton.

MATTEY, D.L. & GARROD, D.R. (1986*a*) Calcium-induced desmosome formation in cultured kidney epithelial cells. *J. Cell Sci.*, **85**, 95.

MATTEY, D.L. & GARROD, D.R. (1986*b*). Splitting and internalisation of the desmosomes of cultured kidney epithelial cells by reduction of calcium concentration. *J. Cell Sci.*, **85**, 113.

MILLER, K., MATTEY, D., MEASURES, H., HOPKINS, C. & GARROD, D.R. (1987). Localisation of the protein and glycoprotein components of bovine nasal epithelial desmosomes by immunoelectron microscopy. *EMBO J.*, **6**, 885.

MOLL, R., COWIN, P., KAPPRELL, H.P. & FRANKE, W.W. (1986). Biology of disease. Desmosomal proteins: new markers for identification and classification of tumours. *Lab. Invest.*, **54**, 4.

MUELLER, H. & FRANKE, W.W. (1983). Biochemical and immunological characterisation of desmoplakins I and II, the major polypeptides of the desmosomal plaque. *J. Mol. Biol.*, **163**, 647.

OSBORN, M. & WEBER, K. (1985). A monoclonal antibody recognising desmosomes: use in human pathology. *J. Invest. Dermatol.*, **85**, 385.

OVERTON, J. (1968). The fate of desmosomes in trypsinised tissue. *J. Exp. Zool.*, **185**, 341.

OVERTON, J. (1974). Cell junctions and their development. *Prog. Surf. Sci. Membr. Sci.*, **8**, 161.

PARRISH, E.P., MARSTON, J.E., MATTEY, D.L., MEASURES, H.I., VENNING, R. & GARROD, D.R. (1990). *J. Cell Sci.*, **96**, 239.

PARRISH, E.P., STEART, P.V., GARROD, D.R. & WELLER, R.O. (1987). Antidesmosomal monoclonal antibody in the diagnosis of intracranial tumours. *J. Pathol.*, **153**, 265.

PASDAR, M. & NELSON, W.J. (1988). Kinetics of desmosome assembly in Madin-Darby canine kidney epithelial cells. Temporal and spatial regulation of desmoplakin organisation and stabilisation upon cell–cell contact. I. Biochemical analysis. *J. Cell Biol.*, **106**, 677.

PAULI, B.U., COHEN, S.M., ALROY, J. & WEINSTEIN, R.S. (1978). Desmosome ultrastructure and the biological behaviour of chemical carcinogen induced urinary bladder carcinomas. *Cancer Res.*, **38**, 3276.

PENN, E.J., HOBSON, C., REES, D.A. & MAGEE, A.I. (1987). Structure and assembly of desmosome junctions: biosynthesis, processing and transport of the major protein and glycoprotein components in cultured epithelial cells. *J. Cell Biol.*, **105**, 56.

POOLE, A.R. (1973). Tumour lysosomal enzymes and invasive growth. In *Lysosomes in Biology and Pathology*, Dingle, J.T. (ed.).

PORTER, K.R. (1954). Observation on the fine structure of animal epidermis. *Proc. Third Conf. Electron Microscopy*, London, p. 281.

QUIGLEY, J.P. (1979). Proteolytic enzymes of normal and malignant cells. In *Surfaces of Normal and Malignant Cells*. John Wiley and Sons: New York.

REYNOLDS, E.S. (1963). The use of lead citrate at high pH as an electron opaque stain on electron microscopy. *J. Cell Biol.*, **17**, 208.

RISAU, W., SAUMWEBER, H. & SYMMONS, P. (1981). Monoclonal antibodies against nuclear membrane of drosophila. *Exp. Cell Res.*, **133**, 47.

RUOSLAHTI, E. & PIERSCHBACHER, M.D. (1987). New perspectives in cell adhesion: RGD and integrins. *Science*, **238**, 491.

SCHINDLER, A.M., AMAUDRUZ, M.A., KOCHER, O., RIOTTEN, G. & GABBIANI, G. (1981). Desmosomes and gap junctions in various epidermoid preneoplastic and neoplastic lesions of the cervix uteri. *Acta Cytol.*, **26**, 466.

SCHMELZ, M., DUDEN, R., COWIN, P. & FRANKE, W.W. (1986). A constitutive transmembrane glycoprotein of Mr. 165,000 (desmoglein) in epidermal and non-epidermal desmosomes. I. Biochemical identification of the polypeptide. *Eur. J. Cell Biol.*, **42**, 177.

SKERROW, C.J. (1986). Desmosomal proteins. In *Biology of the Integument, 2*, Bereiter-Hahn, J., Matoltsy, A.G. & Richards, K.S. (eds) p. 736. Springer-Verlag: Berlin, Heidelberg.

SKERROW, C.J. & MATOLTSY, A.G. (1974*a*). Isolation of epidermal desmosomes. *J. Cell Biol.*, **63**, 515.

SKERROW, C.J. & MATOLTSY, A.G. (1974*b*). Chemical characterisation of isolated epidermal desmosomes. *J. Cell Biol.*, **673**, 524.

STAEHELIN, L.A. (1974). Structure and function of intercellular junctions. *Int. Rev. Cytol.*, **39**, 191.

STEINBERG, M.S., SHIDA, H., GIUDICE, G.J., PATEL, N.H. & BLASCHUK, O.W. (1987). On the molecular organisation, diversity and functions of desmosomal proteins. In *Junctional Complexes of Epithelial Cells*, Bock, G. & Clark, S. (eds) p. 3. Cold Spring Harbor Laboratory: New York.

SUHRBIER, A. & GARROD, D.R. (1986). An investigation of the molecular components of desmosomes in epithelial cells of vertebrates. *J. Cell Sci.*, **81**, 223.

TAKEICHI, M. (1988). The cadherins, cell–cell adhesion molecules controlling animal morphogenesis. *Development*, **102**, 639.

TERRANOVA, V.P., HUJANEN, E.S. & LOEB, D.M. (1986). A reconstituted basement membrane measures cell invasiveness and selects for highly invasive tumour cells. *Proc. Natl Acad. Sci. USA*, **83**, 465.

TOWBIN, H., STAEHELIN, T. & GORDON, J. (1979). Electrophoretic transfer of proteins from polyacrylamide gels to nitrocellulose sheets: procedure and some applications. *Proc. Natl Acad. Sci. USA*, **76**, 4350.

VERSTIJNEN, C.C., ARENDS, J., MOERKERK, P., WARNAAR, S., HILGERS, J. & BOSMAN, F. (1986). CEA specificity of CEA-reactive monoclonal antibodies. Immunochemical and immunocytochemical studies. *Anticancer Res.*, **6**, 97.

VILELA, M.J. (1989). Monoclonal antibodies to desmosomal glycoprotein 1: their contribution to cancer diagnosis and protein structure studies. PhD Thesis, University of Southampton.

VILELA, M.J., PARRISH, E.P., WRIGHT, D.H. & GARROD, D.R. (1987). Monoclonal antibody to desmosomal glycoprotein 1 – a new epithelial marker for diagnostic pathology. *J. Pathol.*, **153**, 365.

WARREN, L. & BUCK, C.A. (1980). The membrane glycoproteins of the malignant cell. *Clin. Biochem.*, **13**, 191.

WATT, F.M., MATTEY, D.L. & GARROD, D.R. (1984). Calcium induced reorganisation of desmosomal components in cultured human keratinocytes. *J. Cell Sci.*, **99**, 2221.

WEINSTEIN, R.S., MERK, F.B. & ALROY, J. (1976). The structure and function of intercellular junctions in cancer. *Adv. Cancer Res.*, **23**, 23.

WEISS, L. & WARD, P.M. (1983). Cell detachment and metastasis. *Cancer Metastasis Rev.*, **2**, 111.

WIERNIK, G., BRADBURY, S., PLANT, M., COWDELL, R.H. & WILLIAMS, E.A. (1973). A quantitative comparison between normal and carcinomatous squamous epithelia of the uterine cervix. *Br. J. Cancer*, **28**, 488.